# Breast Implants: Low Rate of Annual Check-Ups Results in Delayed Presentation of Ruptured Implants

**DOI:** 10.3390/jcm13216545

**Published:** 2024-10-31

**Authors:** Tonatiuh Flores, Celina Kerschbaumer, Christina Glisic, Michael Weber, Klaus F. Schrögendorfer, Konstantin D. Bergmeister

**Affiliations:** 1 Karl Landsteiner University of Health Sciences, Dr. Karl-Dorrek-Straße 30, 3500 Krems, Austria; celina.kerschbaumer@outlook.com (C.K.); christina.glisisc@stpoelten.lknoe.at (C.G.); michael.weber@kl.ac.at (M.W.); klaus.schroegendorfer@stpoelten.lknoe.at (K.F.S.); konstantin.bergmeister@stpoelten.lknoe.at (K.D.B.); 2 Clinical Department of Plastic, Aesthetic and Reconstructive Surgery, University Clinic of St. Poelten, 3100 St. Poelten, Austria; 3 Clinical Laboratory for Bionic Extremity Reconstruction, University Clinic for Plastic, Reconstructive and Aesthetic Surgery, Medical University of Vienna, 1090 Vienna, Austria

**Keywords:** breast implant rupture, awareness, regular check-ups, BIA-ALCL

## Abstract

**Background:** Breast-implant-based reconstruction is one of the most performed procedures in plastic surgery. Despite the high durability of breast implants, various complications are accompanied with prolonged inlay duration, particularly implant rupture. Many aftereffects can be associated with implant rupture, especially siliconoma and BIA-ALCL. Without regular implant check-ups, implant-related issues may remain underrecognized. Here, we analyzed the number of breast implant carriers needing revisions and if patients adhered to annual implant follow-up recommendations. **Methods:** We reviewed 1128 breast procedures at the department of plastic surgery at the University Clinic of St. Poelten between August 1^st^ 2018 and December 31^st^ 2023. Patients were analyzed to see whether regular check-ups of their breast implants were performed. Additionally, implant-related complications were investigated, as well as if they were noticed by implant carriers. **Results:** Only 15.46% of breasts implants were regularly checked at least once a year in our cohort. The remaining 84.54% of patients consulted our department due to pain or aesthetic discomfort without periodical follow-ups. Most implant ruptures (73.8%) were diagnosed in patients consulting acutely due to pain or capsular contraction after an average of 17.36 ± 10.57 years. Routine examination uncovered 26.2% of silent implant ruptures without patients yet complaining of clinical symptoms as early as 15.44 ± 11.17 years. **Conclusions:** Most implant ruptures develop clinical symptoms as an indicator that removal is warranted. However, only regular follow-ups can identify implant complications several years earlier and possibly reduce severe sequalae such as BIA-ALCL. This highlights the significance and necessity of annual breast implant controls by surgeons and radiologic imaging to prevent devastating implant-associated aftereffects.

## 1. Introduction

Breast-implant-based operations are the most performed procedures in plastic surgery on the female breast [1,2]. They account for approximately 57% of breast surgeries worldwide [2,3,4]. In 2022, nearly 2 million breast-implant-based surgeries were performed, with more than 35 million implant carriers globally [1,2,5,6,7,8].

Breast implants are highly durable medical devices, yet their change or removal is recommended approximately every 10 years [8,9]. Even though implants are considered safe, they may entail various complications [10,11,12], most of which are easily recognized by patients as they are frequently associated with pain or breast deformity [12,13,14]. However, breast implants are often damaged unknowingly, potentially leaving women exposed to undetected implant-associated threats [9,12,15,16,17,18].

Implant rupture is one of the most prevalent implant complications, yet it often occurs silently and is a significant contributor in the pathogenesis of BIA-ALCL (breast-implant-associated anaplastic large cell lymphoma) [19,20]. This is a rare, yet severe lymphoma exclusively linked to breast implants with potentially lethal progression [8,16,20,21,22,23,24,25,26]. Its manifestation is on average after 8 to 10 years, and thus, its onset might commence long before its clinical appearance [20]. As of the low incidence of BIA-ALCL, the awareness of especially silent long-term implant complications remains poor among implant carriers [6,7,20,27]. To prevent these severe implant-associated complications, regular screening for implant failure is imperative [28,29,30,31]. Although surgeons encourage women to undertake annual or biennially routine check-ups, unfortunately, these recommendations are often neglected [32,33]. In this paper, we aimed to investigate the rate of periodical breast implant examinations among women who needed revision at our department.

## 2. Materials and Methods

In this study, we reviewed patients undergoing breast implant revision due to alleged implant-associated issues at the Clinical Department for Plastic, Aesthetic and Reconstructive Surgery at the University Hospital St. Poelten, between August 1^st^ 2018 and December 31^st^ 2023. The study was conducted as a retrospective single center study. Ethical approval was obtained from the local institutional review board at the Karl Landsteiner University of Health Sciences, Krems (reference number: ECS 1005/2020). The revised factors included the patients’ age at surgery, BMI, breast implant inlay duration, whether patients had regular checks of their implants or not, reasons for consultation at our outpatient clinic and duration of hospital stay.

All statistical analyses were performed using IBM SPSS Statistics for Windows version 29 (© IBM, Armonk, NY, USA). The metrical data are described using mean ± SD in case of normal distributed data or median [min; max] when skewed. To compare groups, Mann–Whitney U-tests were used due to skewed data. A *p*-value equal or below 0.05 was considered statistically significant. Due to the small sample size, no multiplicity corrections were performed to avoid an increased error of the second type.

The patients included were women who experienced breast implant reconstruction due to breast cancer or due to cosmetic reasons in their medical history. Surgical procedures included implant removal or implant to implant change. Patients undergoing prior breast implant reconstruction or breast implant revision at our institution were regularly followed-up for at least one year.

## 3. Results

**Figure 1:**
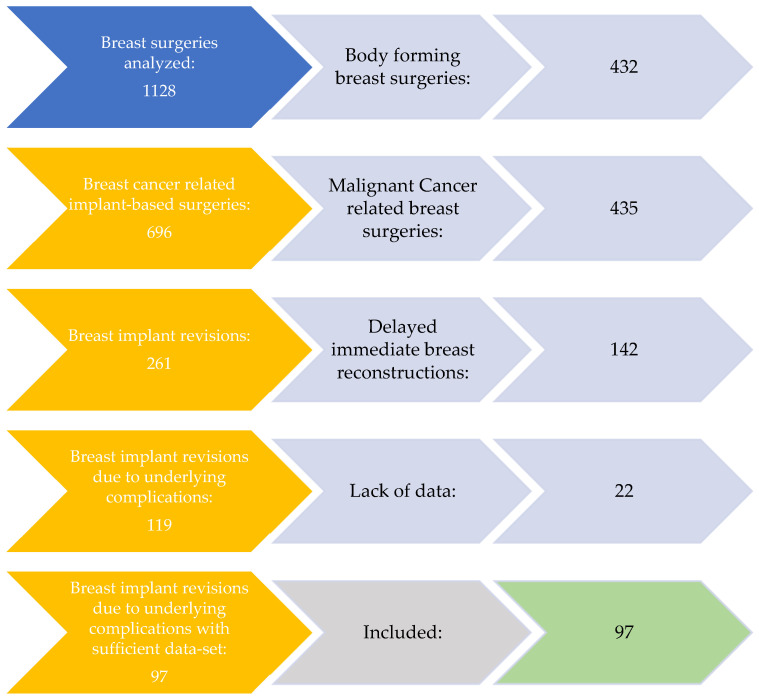
Organigramme of patient selection for study inclusion.

In total, 1128 breast surgeries were identified within our study period (Figure 1). This included 432 body contouring procedures and 435 malignant-cancer-related breast surgeries. Of the remaining 261 implant-based revisions, 142 surgeries were delayed-immediate reconstruction due to breast cancer. Thereof, 119 breast implant revisions were performed due to underlying complication. Here, 22 revisions were excluded due to lack of data. Finally, 97 breast implants in 56 patients met our criteria and were included in our study. Sub-analyses were performed based on implant indication (cosmetic or reconstructive) and indication for revision: implant failure or implant revisions due to capsular contraction, breast deformity (e.g., waterfall deformity, recurrent ptosis) or pain.

### 3.1. Demographics

The mean patient age at surgery was 51.980 years ± 15.984 years, ranging from 21 to 83 years (Table 1). The mean BMI was 24.30 kg/m^2^ ± 4.900 kg/m^2^, varying from 17.1 kg/m^2^ to 41.5 kg/m^2^. The mean duration of surgery at revision was 111.61 min ± 64.42 min, varying from 21 to 285 min. The mean hospital stay was 4.25 days ± 2.213 days, ranging from 1 to 10 days.

**Figure 2:**
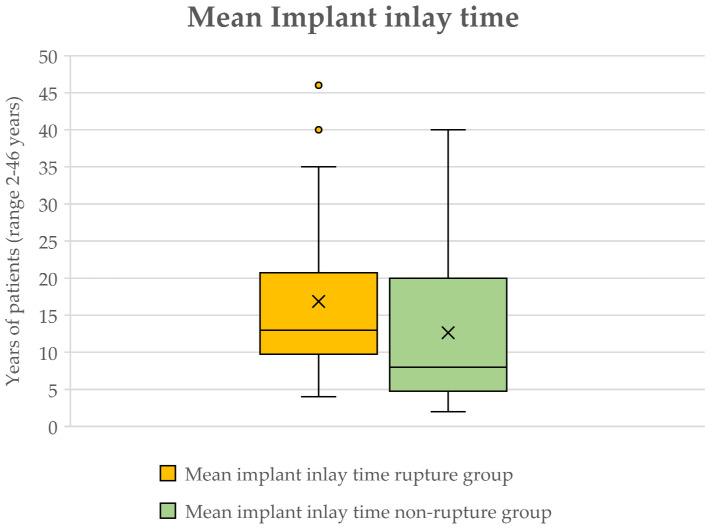
Boxplot of mean implant inlay time among groups. Implant rupture group is depicted in orange and non-rupture group in green. Note that the time point of implant revision does not differ significantly among groups. The “x” marks mean implant inlay time of 16.88 years in the rupture group and 12.64 years in the non-rupture group. Outliers can be seen in the rupture group marked as dots.

The mean implant inlay time was 15.21 years ± 10.804 years, ranging from 2 to 46 years. The implant inlay time in the rupture group was 16.88 years ± 10.970 years, ranging from 4 to 46 years and 12.64 years ± 10.520 years, ranging from 2 to 40 years in the non-rupture group (Figure 2).

**Figure 3:**
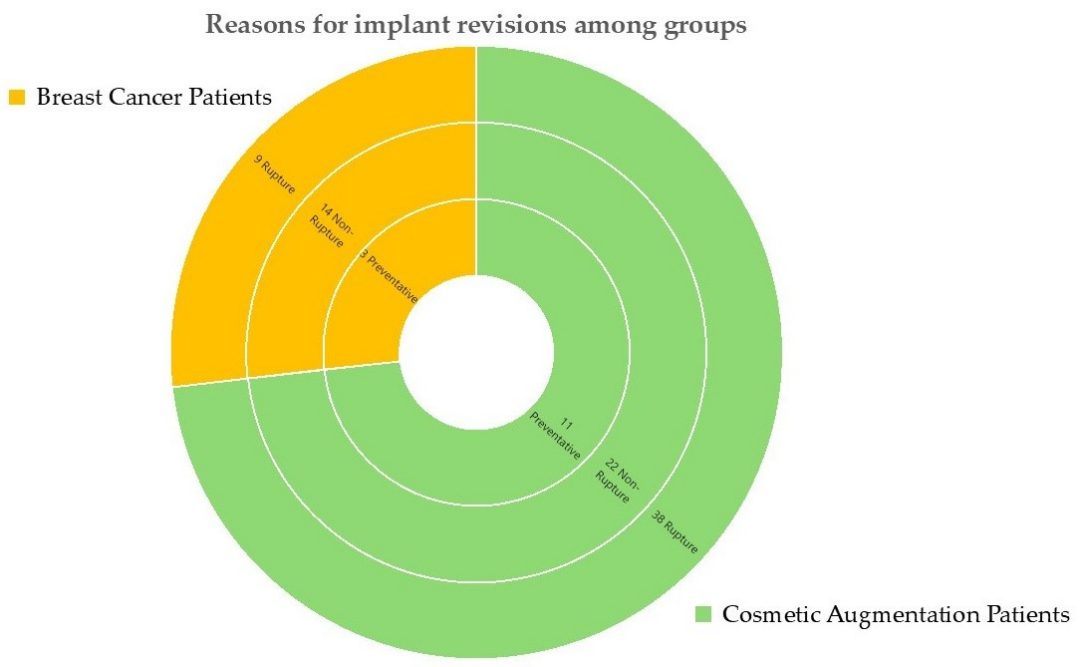
Indications for breast implant revisions among groups. Patients with history of breast cancer are displayed in yellow; patients with cosmetic augmentation prior to revision are displayed in green.

The mean hospital stay was 4.85 days (±2.377) in rupture patients and 3.32 days (±1.550) in the non-rupture group. The underlying cause for breast implant reconstruction prior to revision was cosmetic breast augmentation (in external facilities) in 71 (73.2%) breasts and cancer-related breast reconstruction in 26 (28.80%) breasts (Figure 3).

Overall, 97 breast implants revision occurred, thereof 47 (48.45%) were removed due to implant rupture and 36 (37.12%) due to capsular fibrosis, deformities or pain. The remaining 14 (14.43%) prostheses were removed as prophylactic measure or for symmetry reasons (Figure 3).

Only 15 (15.46%) out of 97 breast implants were regularly checked via radiologic imaging and physical examination once a year. Here, 10 (66.66%) breast implant ruptures were detected.

### 3.2. Implant Rupture Group

**Figure 4:**
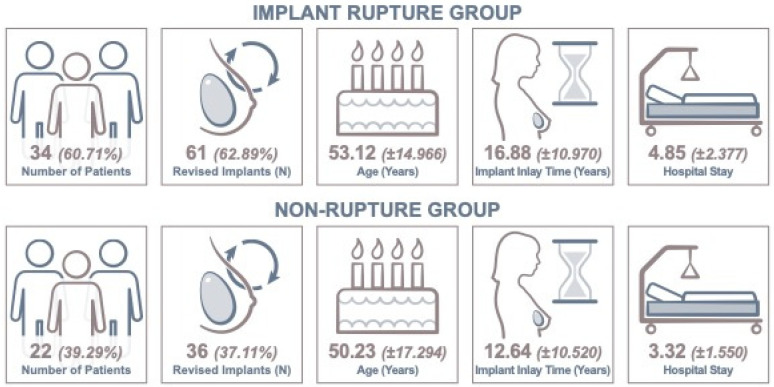
Key data chart of included patients and study findings.

Overall, 47 (48.45%) ruptured implants in 34 (60.71%) patients were removed. The mean age at surgery was 53.12 years ± 14.966 years, ranging from 29 to 83 years (Table 1). The mean BMI was 25 kg/m^2^ ± 5 kg/m^2^, varying from 17.1 kg/m^2^ to 41.5 kg/m^2^. Overall, the implant inlay time was 16.88 years ± 10.970 years in this group, ranging from 4 to 46 years (Figure 4).

**Figure 5:**
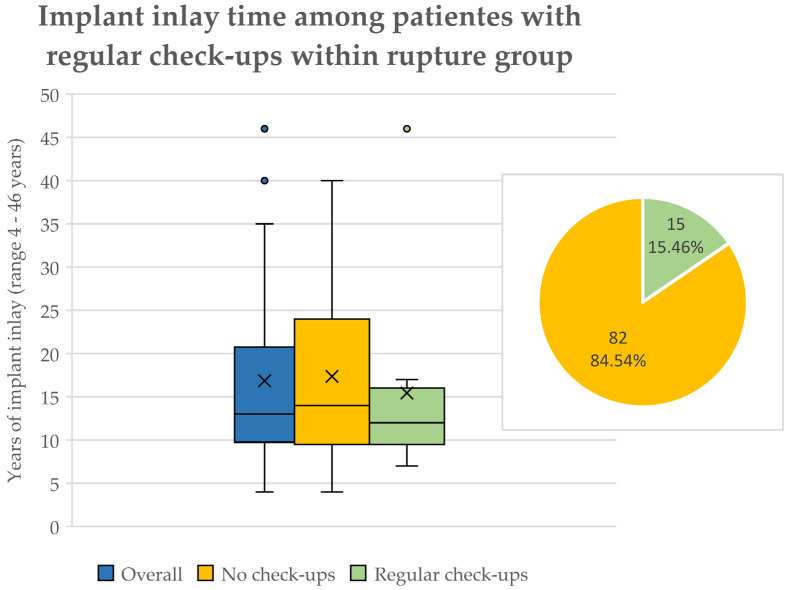
Boxplot of breast implant inlay time in patients experiencing breast implant rupture. Implant inlay time was lower in patients regularly experiencing breast implant check-ups, yet not significantly. Overall inlay time portrayed in blue, patients without check-ups are seen in orange, and patients with regular check-ups are seen in green. The *X* marks the mean implant inlay time of 15.25 years in the group with regular check-ups and 17.38 years in the non-check-up group. Outliers are depicted as dots. Percentages of regularly checked breast implants can be seen in the top right corner. Regularly checked implants are depicted in green (15, 15.46%). Patients without regular check-ups are seen in orange (82, 84.54%).

The mean implant inlay time in patients regularly undergoing check-ups was 15.25 years ± 9.42 and 17.38 years ± 11.53 in patients without follow-ups (Figure 5).

**Figure 6:**
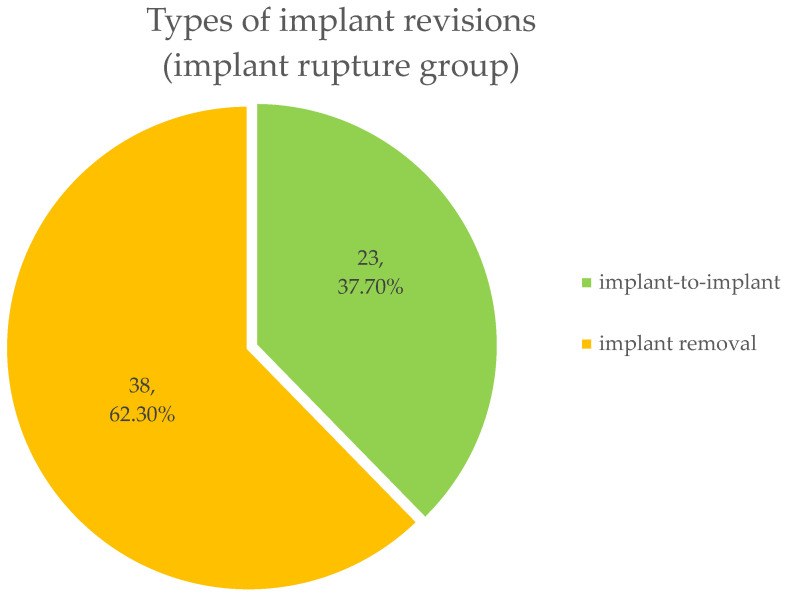
Different types of implant revisions. 62.30% of implants were removed. Residual 37.7% of implants were exchanged.

The mean duration of surgery was 120.6 min ± 66.25 min, varying from 24 min to 285 min in case of heavenly silicone-matted implant cavity and bilateral surgery. The mean hospital stay overall was 4.85 days ± 2.377 days, ranging from 2 to 10 days. Patients in this group underwent implant-to-implant change in 24 (39.34%) cases. The residual 37 (60.66%) cases were implant removals (Figure 6).

Only 15 (15.46%) implants in 9 (16.07%) patients were regularly checked via either MRI or sonography and physical examination once a year (Figure 5). Here, 9 (9.28%) implants did not show any concomitant symptoms (breast deformity, pain, etc.).

### 3.3. Non-Rupture Group

In this group, none of the 22 patients experienced breast implant rupture. The mean age at surgery was 50.23 years ± 17.294 years overall, ranging from 21 to 80 years (Table 1). The mean BMI was 24.01 kg/m^2^ ± 4.222, varying from 17.1 kg/m^2^ to 32 kg/m^2^. The mean implant inlay time was 12.64 years ± 10.280 years, ranging from 2 to 40 years. The mean duration of surgery was 97.73 min ± 58.839 min, varying from 21 to 223 min in case of capsulectomy and bilateral surgery. The mean hospital stay was 3.32 days ± 1.520 days, ranging from 1 to 8 days.

**Figure 7:**
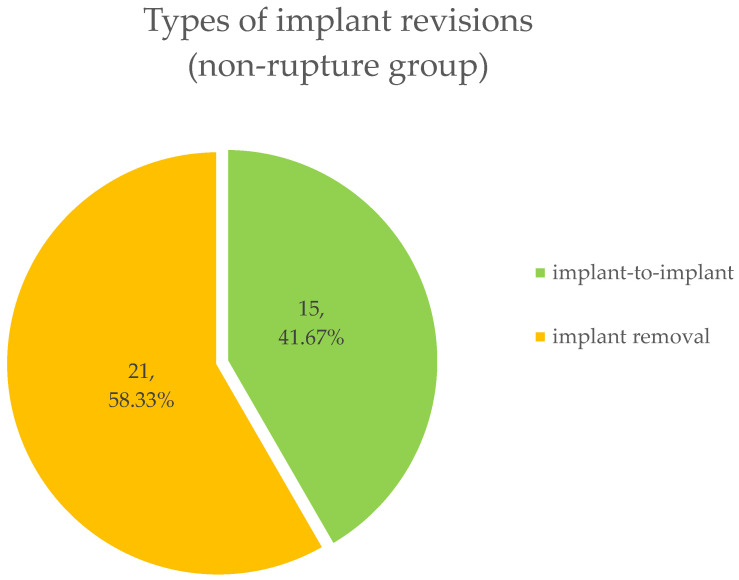
Different types of implant revisions. 58.33% of implants were removed. Residual 41.67% of implants were exchanged.

In this group, 36 (37.11%) implant revisions were performed. None of the patients had breast implant check-ups on a regular basis, and no implant ruptures occurred in this group. The reasons for removal were pain (11 implants, 30.56%), capsular contraction (22 implants, 61.12%), breast cancer recurrence (1 implant, 2.77%) and painless capsular contraction (2 implants, 5.55%). At surgery, implant-to-implant change was performed in 15 (41.67%) cases and removal only in 21 cases (58.33%) (Figure 7).

### 3.4. Statistical Analyses

The mean inlay time in women with implant rupture was 16.88 years (±10.970), whereas women without implant rupture showed a mean inlay time of 12.64 years (±10.520). When conducting a Mann–Whitney U-Test for non-normally distributed variables regarding implant inlay time between our groups, no statistical significance could be observed (*p* = 0.071). Yet, a strong tendency toward longer implant inlay time in women with implant rupture can be observed (Table 2).

The mean time of surgery was 120.6 min (±66.250) in case of rupture and 97.73 min (±58.839) in case of non-rupture. Mann–Whitney U-Testing showed no significant difference in the duration of surgery between rupture and non-rupture patients (*p* = 0.205) (Table 2).

The patients experiencing implant rupture showed a mean hospital stay of 4.85 days (±2.377). The women without rupture were admitted for 3.32 days (±1.550) on average. This difference could be proven to be statistically significant between our groups, using the Mann–Whitney U-Test (*p* = 0.016), demonstrating that women experiencing implant rupture required more convalescence time after surgery (Table 2).

Furthermore, we performed the Mann–Whitney U-Test to analyze patients with and without regular check-ups. Here, no statistical significance could be observed at implant inlay time (*p* = 0.735). Moreover, no significant difference could be seen regarding duration of surgery (*p* = 0.347) or hospital stay (*p* = 0.618) (Table 3).

## 4. Discussion

Breast implants are essential medical devices in aesthetic and reconstructive surgery [34,35,36]. Their main purpose is to augment or restore the female breast shape and enhance women’s quality of life [34,36,37,38,39]. Nevertheless, once implanted, breast implants entail a variety of drawbacks in addition to showing relatively low complication rates [4,16,40,41,42,43,44]. Many implant-associated issues occur silently or progress slowly and are, therefore, often noticed late by patients with increasing pain [45,46,47,48,49]. Mild capsular contraction, dislocation or even rupture are often not recognized at all by patients and might, therefore, cause further aggravation [13,32,33,50,51,52]. Additionally, many women consult surgeons only after troublesome incidents, such as trauma, severe aesthetic discomfort or pain, which might take place years after the initial implant issue [53,54,55,56].

In addition to international recommendations for regular implant changes approximately every 10 years, the exact time point of revision remains incalculable [4,8,9,14,57]. As many implant ruptures occur silently, the exact timepoint of revision remains unnoticed. [9,12,15,20,45]. Only if it clinically manifests the indication for revision appears evident [12,13,20,23,48,58,59,60].

**Figure 8:**
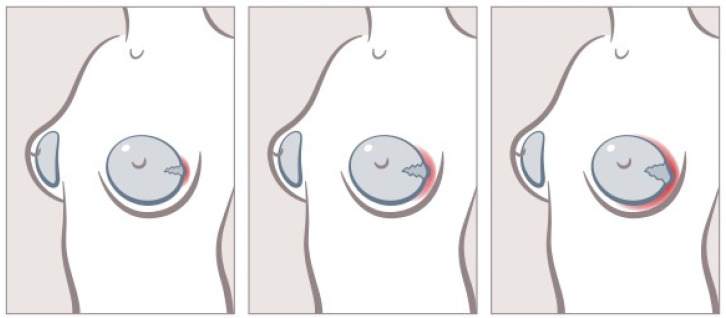
Inflammatory process encapsulating the breast implant. Longer exposure entails increased peri-implantary inflammation, further damaging the implant shell. This vicious circle can be sighted at an early stage in case of regular breast implant check-ups once a year.

A potentially severe implant complication is rupture of the implant shell [9,11,30,49,61]. Here, silicone gel filling is exposed, provoking inflammation and chronic cellular stress on the human body [20,41]. Such immunological mechanisms could already be linked to the pathogenesis of BIA-ALCL, a rare yet potentially fatal malignancy [20,27,62,63,64,65]. Thus, these patients may already undergo the immunological responses following silicone exposure and thus eventually progress to BIA-ALCL tumorigenesis (Figure 8). 

**Figure 9:**
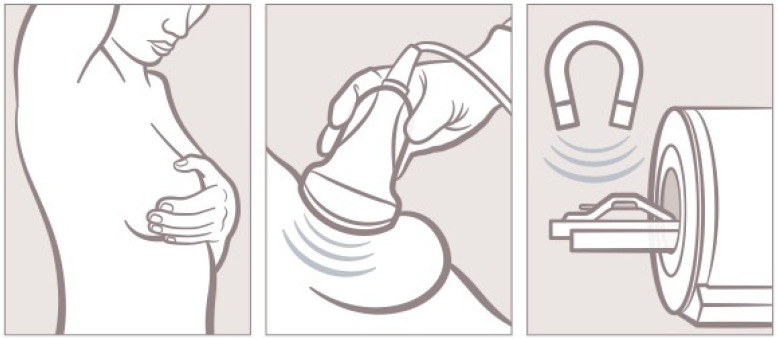
Pictography of possible tools for regular implant control. Self-examinations are easily performed frequently. Ultrasound examinations of the breast are to be conducted at least annually. MRI examination can be performed additively or carried out directly for implant follow-up.

Yearly breast implant follow-ups with physical examination by surgeons and radiological imaging (ultrasound, MRI) are thus strongly advised to identify problems early on [66]. This is especially required in women without apparent signs of implant complications regardless of the initial indication of breast implant insertion [66]. Here, mild capsular contraction, or even implant rupture, could be identified earlier and, in the case of silent implant issues, treated sooner. Whereas cosmetic augmentation patients ought to be followed-up with yearly using physical examinations and at least biannually through radiographic imaging as stated by the FDA, self-examinations additionally are a powerful tool in recognizing potential implant complications [66,67,68,69,70]. Auto-palpation of the implant can easily be performed on a daily basis by women themselves. Thereby, changes in implant shape, pliability or position can better be annotated, and actions can be taken (Figure 9). Breast cancer patients are to be checked even more frequently [31,66,71]. After cancer removal, patients undergo physical examinations every 3 months in the first year, every 6 months in the second and third year and annually after four years in case of low-risk profile in our clinic. Radiographic imagining is only conducted in case of noticeable implant complication. Additionally, breast cancer patients are deeply encouraged to simultaneously consult our outpatient clinic at the time of oncologic follow-up; this recommendation is often ignored in case of non-recurrence. This might be due to the fact that cancer-survivors in our cohort tend to assume the implant is unharmed in case of inconspicuous oncologic control. Yet only if paired with yearly ultrasound or MRI can implant assessment sufficiently be performed parenthetically [44,72,73]. In case of a high-risk profile, patients are followed-up every 3 and 6 months in the first two years and every 6 months after the third year. Here, ultrasound and MRI are performed every 6 months. Unfortunately, precise follow-up modalities for breast implant monitoring are not uniform globally. Whereas radiologic imaging is feasible in Europe as health insurances cover a broad spectrum of medical examinations, it is more expensive in the U.S. [74,75,76,77]. Furthermore, necessary examinations are not easily conductible in low-income countries [78], leaving the generalization of implant surveillance challenging [31,44,66,77,79,80].

Nonetheless, regular implant examinations are of utmost importance as the implants’ durability is not predictable and problems may therefore occur early on or after several years [4,8,9,28,29,30]. Despite this need, patients rarely undergo routine control of their implants due to various reasons [8,9]. Furthermore, follow-up behavior differs significantly among patients [81]. Most women who experienced cosmetic breast augmentation tend to omit annual follow-ups beyond regular postoperative inspection if recovery proceeds plainly [66]. This was especially seen in younger women [32]. Contrarily, women of advanced age might focus more actively on possible health issues, consequently consulting surgeons more often [82,83].

Patients undergoing breast reconstruction due to breast cancer are also scheduled more extensively for check-ups [28,38,80] not only for physical examination and planned MRI but also for long-term follow-up within the cancer recurrency period [84,85,86]. Although we did not experience any breast cancer recurrence within our cohort, cancer patients consulted our department more commonly yet too seldomly [87,88].

Moreover, prominent implant-associated complications, e.g., capsular contraction, pain or dislocation, are more noticeable for women. Thus, implant check-ups are seen more often in this population, whereas implant rupture can remain silent over years, therefore, not seeming to entail the need for control examinations [89]. Additionally, because gel implants are mainly used in Austria, rupture does not come with implant deflation as seen in saline implants [90]. Finally, patients also missed follow-ups at our institution during the SARS-CoV-2 Pandemic. Due to major shutdowns within the Austrian health system, only emergency visits were performed by patients. Furthermore, many patients refused to visit our outpatient clinic unless it was inevitable.

As of the variety of factors of nonadherence regarding breast implant follow-up, surgeons performing breast reconstruction or cosmetic augmentation should emphasize postoperative aftercare more vehemently [31,79]. Breast implant registers should be implemented in a more generalized manner to efficiently reach breast implant carriers and to sight possible complications sooner. Moreover, information brochures handed out after implant surgery and patient questionnaires at regular check-ups might support women in gaining implant-awareness [91,92]. Although we encourage our patients to regularly perform breast implant check-ups, only a few women adhere to our recommendations.

The particularly low number of regular follow-ups is thus evident in our study, as only 15.46% of implants were checked annually via radiographic controls and physical examination. In these cases, implant rupture was readily identified and, thus, silicone exposure duration minimized. Consequently, also, the risk for any possible long-term aftereffects such as BIA-ALCL is reduced. Although this could not sufficiently be proven statistically in our study as women with ruptured implants merely showed a strong tendency of longer implant inlay time (*p* = 0.071), this trend depicts a justification of regular implant check-ups. To fully substantiate our assumption, increased sample sizes and prospective study multicentricity is necessitated. However since regular controls are so rarely performed, many patients may already have broken devices [45].

Additionally, the ancillary burden laid on women with ruptured devices could be demonstrated in the past [93]. As revision tends to take longer in case of implant failure, due to the necessity for adequate cavity cleansing, patients may endure increased postoperative pain, bleeding and even elevated drainage fluid volume. This could also be observed within our cohort, as patients with ruptured implants experience longer hospital stays (*p* = 0.016).

Unfortunately, our study faces some limitations. Our relatively low sample size limits generalization. Furthermore, statistical analyses on demographics, lifestyle and socioeconomic factors ought to be delineated in additional studies. Because this research focuses on the follow-up rates of women in need of implant revision, our study holds a certain bias in terms of recall and patient selection. Additionally, patients and physicians were not blinded, as the reason for consultation was due to underlying complication. Although women experiencing implant-based procedures at our institution are followed up regularly within the first years, many women refused further check-ups if no implant issue was obvious. Nonetheless, due to the significance of this topic, prospective multicenter studies are needed to better reflect the causes of neglected implant follow-ups. Breast implant carriers might benefit from potential conclusions drawn from larger scale studies and enhance their quality of life after breast implant surgery [66].

Since primary prevention is the initial step in health maintenance and in detecting and counteracting disease progression, annual implant check-ups are key to early complication prevention [29,30]. Efficient and safe procedures, such as sonography or MRI and physical examination by surgeons, portray the cornerstone in contracepting subclinical implant-related sequalae such as malignancies, notably BIA-ALCL [20,21,28,66].

## 5. Conclusions

Our findings highlight the need for regular breast implant controls. They are of utmost importance in preventing short- and long-term consequences as many commence long before patients experience aesthetic discomfort or pain. As annual breast implant screening through radiologic imaging and physical examination by surgeons are the only modalities to detect silent implant issues, especially implant rupture, their significance needs to be emphasized among women. We conclude that knowledge of implant complications and the necessity of strengthening implant awareness among women need to be invigorated.

## Figures and Tables

**Table 1:** Baseline characteristics of patients undergoing breast implant revision.

Patient Characteristics	Rupture Group	Non-Rupture Group	Total
Age (years)	53.12 (±14.966)	50.23 (±17.294)	51.98 (±15.984)
BMI (kg/m^2^)	25 (±5.000)	24.01 (±4.222)	24.30 (±4.900)
Revised implants (N)	61 (62.89%)	36 (37.11%)	97
Ruptured Implants (N)	47 (100%)	0 (0%)	47
Non-Ruptured Implants (N)	14 (28%)	36 (72%)	50
Breast Cancer Patients (N)	8 (47.10%)	9 (52.90%)	17
Cosmetic Patients (N)	26 (66.67%)	13 (33.33%)	39
Implant Inlay Time (years)	16.88 (±10.970)	12.64 (±10.520)	15.21 (±10.804)
Duration of Surgery (minutes)	120.6 (±60.220)	97.73 (±67.300)	111.61 (±64.420)
Hospital Stay (days)	4.85 (±2.377)	3.32 (±1.550)	4.25 (±2.213)

**Table 2:** Mann–Whitney U-Test between implant rupture and non-rupture group. A strong tendency of longer inlay time in women with ruptured implants can be seen (*p* = 0.071), concluding, that women with ruptured implants tend to undergo implant revision later. Regarding duration of surgery, no significant difference is seen between the groups. Finally, women with ruptured implants need longer recovery time after surgery, as can be seen in the statistically significant *p*-value (*p* = 0.016).

Mann–Whitney U-Test									
	Rupture				Non-Rupture				
	Min	Max	Mean	STD	Min	Max	Mean	STD	*p*-Value
Implant Inlay Time	4	46	16.88	10.97	2	40	12.64	10.52	0.071
Duration of Surgery	24	285	120.59	67.30	21	223	97.73	60.22	0.205
Hospital Stay	1	10	4.85	2.40	1	8	3.32	1.55	0.016

**Table 3:** Mann–Whitney U-Test between women with and without regular check-ups. No statistical significance can be seen between the groups, either regarding implant inlay time, duration of surgery nor hospital stay.

Mann–Whitney U-Test									
	Check-Ups				No Check-Ups				
	Min	Max	Mean	STD	Min	Max	Mean	STD	*p*-Value
Implant Inlay Time	4	33	15.25	9.42	5.83	46.00	17.38	11.53	0.735
Duration of Surgery	37	202	96.30	51.2	24	285	128.10	70.6	0.347
Hospital Stay	2	10	4.60	2.6	1	9	4.9	2.4	0.618

## Data Availability

All the data analyzed during the current study are available from the corresponding author on reasonable request.
